# Treatment trends for muscle-invasive bladder cancer in Germany from 2006 to 2019

**DOI:** 10.1007/s00345-022-04017-z

**Published:** 2022-04-29

**Authors:** Luka Flegar, K. Kraywinkel, A. Zacharis, C. Aksoy, R. Koch, N. Eisenmenger, C. Groeben, J. Huber

**Affiliations:** 1 Department of Urology, Philipps-University Marburg, Baldingerstr., 35043 Marburg, Germany; 2 National Center for Cancer Registry Data, Robert Koch Institute, Berlin, Germany; 3 Department of Medical Statistics and Biometry, TU Dresden, Dresden, Germany; 4 Reimbursement Institute RI Innovation GmbH, Hürth, Germany

**Keywords:** Muscle-invasive bladder cancer, Radical cystectomy, Centralization, Population-based analysis, Health services research

## Abstract

**Purpose:**

To examine national treatment trends of muscle-invasive bladder cancer (MIBC) in Germany with a special focus on radical cystectomy (RC).

**Patients and methods:**

Population-based data were derived from the nationwide hospital billing database of the German Federal Statistical Office and institution-related information from the reimbursement.INFO tool based on hospitals’ quality reports from 2006 to 2019. Additionally, we used the German National Center for Cancer Registry data to analyze all cases of bladder cancer with stage ≥ T2 who received RC, chemotherapy, radiation therapy or a combination from 2006 to 2017.

**Results:**

The annual number of RC cases in Germany increased by 28% from 5627 cases in 2006 to 7292 cases in 2019 (*p* = 0.001). The proportion of patients undergoing RC remained constant at about 75% in all age groups between 2006 and 2017 (*p* = 0.3). Relative to all performed RC, the proportion of patients > 75 years increased from 25% in 2006 to 38% in 2019 (*p* = 0.03). The proportion of patients receiving a combination of RC and chemotherapy increased from 9% in 2006 to 13% in 2017 (*p* = 0.005). In 2006, 8 of 299 urology departments (2.7%) performed more than 50 RCs per year, which increased to 17 of 360 (4.7%) in 2019. In 2019, 107 departments (29%) performed 25–49 RCs and 236 (66%) departments performed < 25 RCs.

**Conclusion:**

In Germany, three out of four patients with MIBC receive RC and the proportion of patients > 75 years is increasing. The combination of surgery and chemotherapy is increasingly used. With overall increasing case numbers, there is a slight tendency towards centralization.

**Supplementary Information:**

The online version contains supplementary material available at 10.1007/s00345-022-04017-z.

## Introduction

Urinary bladder cancer is the ninth most common cancer worldwide, with approximately 3% of all new cancer diagnoses and responsible for 2% of all cancer deaths [1, 2].

Current evidence-based guidelines recommend radical cystectomy (RC) with pelvic lymphadenectomy as the primary most effective treatment approach for localized muscle-invasive or recurrent high-grade non-muscle-invasive urothelial carcinoma of the urinary bladder [3, 4]. Several studies showed a recurrence-free survival of 60–70% after 5 and 60% after 10 years for patients undergoing RC [3, 5].

RC is an extensive surgical and reconstructive procedure involving the removal of the complete urinary bladder and adjacent pelvic organs [6]. Due to its complexity, the surgical intervention is associated with a prolonged postoperative recovery as well as increased morbidity and mortality rates. Especially older patients with significant comorbidities are at a disadvantage [7]. Therefore, alternative therapy approaches including chemotherapy as well as radiation therapy (RT) and the combination of both as radiochemotherapy have been introduced for surgical unfit patients or those with a special focus on bladder preservation and quality of life (QoL). Neoadjuvant chemotherapy is associated with a survival benefit but uptake of chemotherapy before RC for muscle-invasive urinary bladder cancer (MIBC) has been low and slow [8]. Furthermore, functional results after RC are of great importance and the loss of the bladder with urinary diversion means a significant restriction of the postoperative QoL.

Previous studies have investigated the use of RC for the treatment of MIBC and showed a serious underutilization of RC especially in older and frail patients in the USA [9]. To our knowledge, there are no current population-based studies examining the utilization of RC for MIBC in Germany.

Consequently, our aim was to evaluate treatment trends for MIBC with a special focus on the utilization of RC in Germany from 2006 to 2019.

## Patients and methods

### Databases

Since there is no large comprehensive cancer-related database available in Germany, we analyzed data from three different sources. We queried the German National Center for Cancer Registry Data, the German hospitals’ quality reports and the nationwide hospital billing database of the German Federal Statistical Office (Destatis database). Table 1 presents an overview of the queried databases. The Destatis database contains billing data and was used for the analysis of all surgical procedures. The quality reports are based on the same data set but allowed the geographical localization of respective hospitals. The German National Center for Cancer Registry Data was supplemented for nationwide incidence estimation and assessment of multimodal therapies.

**Table 1:** Overview of the queried databases

Source data	German Federal Cancer Registries	German nationwide hospital billing data	
Database	German National Center for Cancer Registry Data	Nationwide hospital billing database of the German Federal Statistical Office (Destatis database)	German hospitals’ quality reports (reimbursement.INFO tool)
Reported data	Age and sex Diagnosis code Type of treatment (surgery, chemotherapy, radiation therapy)	Age and sex Diagnosis code Type of surgery and approach Hospital characteristics (teaching status, size, annual surgery caseload, approaches for surgery)	Age and sex Type of surgery Hospital characteristics (teaching status, annual surgery caseload) Geographical localization of respective hospitals
Number of patients	24.534	97.176	88.464 (years 2007, 2009 and 2011 missing)
Proportion of the population	31%	100%	100%

The German National Center for Cancer Registry Data (ZfKD) at the Robert Koch Institute in Berlin contains information on malignant diseases in Germany. It annually derives its data from the German federal cancer registries and processes it for analysis on a national level [10].

We identified all patients with MIBC in combination with received treatment modalities [surgery (RC), chemotherapy, RT and a combination of those as well as no reported therapy] between 2006 and 2017. For our analysis, six federal states (Bavaria, Brandenburg, Mecklenburg-Vorpommern, Saxony, Saxony-Anhalt and Thuringia) provided data, which met the inclusion criteria (< 10% of patients with no/missing treatment information). Thus, 24.534 patients (7.283 women) with MIBC were included in the analysis. Therefore, we were able to cover around 31% of the German population for the present study.

The annual caseload of RC from 2006 to 2019 was analyzed by using the reimbursement. INFO tool (Reimbursement Institute, Hürth, Germany) based on hospitals’ quality reports. Since 2005, German hospitals have been required by law to provide detailed information in quality reports. For data protection reasons, interventions that are performed only 1–3 times per year in the hospital are anonymized in quality reports and presented with case number 1 for the present work. Combinations of ICD codes with OPS codes are prohibited. Therefore, differentiation of RC for malignant or benign disease is impossible. We used OPS codes “5-576.2, 5-576.3, 5-576.4, 5-576.5, 5-576.6, 5-576.7, 5-687” representing RC.

The identified hospitals were classified for RC caseload. Map displays were performed using “EasyMap 11.1 Standard Edition” (Lutum + Tappert DV-Beratung GmbH, Bonn, Germany).

Reimbursement of inpatient treatment is regulated with diagnose-related groups (DRG) since 2004 in Germany. The German Federal Statistical Office (Destatis) collects part of these data. We included patients with the diagnosis of bladder cancer (ICD code “C67.0–C67.9”) as well as OPS codes “5-576.2, 5-576.3, 5-576.4, 5-576.5, 5-576.6,5–576.7, 5-687” for RC. Surgical approach was defined as laparoscopic (OPS-codes 5-576.01–5-576. × 1), robotic-assisted (OPS-code 5-987) or open for the remaining cases without additional codes. The relevant patient cohort was identified similar to our previously described method [11].

### Statistical analysis

Data was presented by absolute and relative frequencies. Linear regression models were implemented to detect trends over time. We defined *p* < 0.05 to indicate statistical significance. We used SPSS 27.0 (IBM corp., Armonk, NY, USA) for our statistical analysis.

### Ethics statement

Our study was performed in accordance with the Declaration of Helsinki in its latest version. All data was completely de-identified and derived from established databases and cancer registries. Therefore, an additional ethics statement was not required.

## Results

**Fig. 1:**
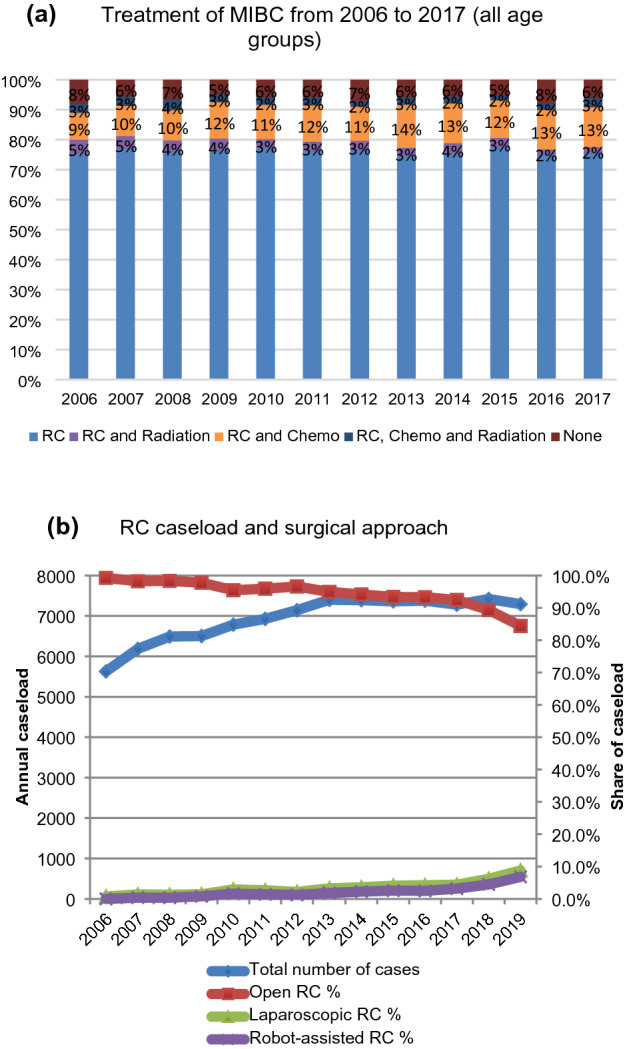
**a** Share of treatment trends for muscle-invasive bladder cancer in Germany from 2006 to 2017 in percent for all ages (0% from 2006–2017: Chemotherapy, RT and Chemo, RT) ( Source: German National Center for Cancer Registry Data) and **b** surgical approach and an absolute number of RC cases from 2006 to 2019 [Source: Nationwide hospital billing database of the German Federal Statistical Office (Destatis database)]

From the German National Cancer Registry Data, we included 24.534 patients with MIBC between 2006 and 2017. Figure 1 shows the share of each treatment modality among all analyzed cases and the different surgical approaches for RC. The share of patients receiving RC remained constant at 75% (*p* = 0.3). The share of patients receiving chemotherapy (*p* = 0.12) or RT (*p* = 0.09) alone remained constant during the study period. The share of patients receiving a combination of chemotherapy and surgery increased from 2006 to 2017 from 9 to 13% (*p* = 0.005). The share of patients receiving no treatment decreased from 2006 to 2017 from 8 to 6% (*p* = 0.01). The yearly number of open RC declined from 99.3 to 84.4% (*p* < 0.001). The laparoscopic approach increased from 0.7 to 8.8% (*p* < 0.001) and the percentage of robotic RC increased from 0 to 6.8% (*p* < 0.001).

Figure 2 (online supplement) represents shares of each treatment for different age groups. For patients younger than 60 years, the share of RC remained constant at 70% (*p* = 0.3). Combination of RC and chemotherapy increased from 17% in 2006 to 21% in 2017 (*p* = 0.03). For patients between 60 to 79 years, the share of RC increased slightly from 73% in 2006 to 76% in 2017 (*p* < 0.001). Combination of RC and chemotherapy increased from 10% in 2006 to 15% in 2017 (*p* = 0.002). For patients older than 80 years of age, the share of RC remained stable around 80% (*p* = 0.08). The second most common combination for this age group was RC with RT with a constant share of 6% (*p* = 0.08). RC in combination with chemotherapy increased from 2% in 2006 to 4% in 2017 (*p* = 0.008).

The annual number of RC cases in Germany for patients with MIBC increased by 28% from 5,627 cases in 2006 to 7,292 cases in 2019 (*p* = 0.001). Relative to all performed RCs, the proportion of patients > 75 years increased from 25% in 2006 to 38% in 2019 (*p* = 0.03). In 2006, 8 of 299 urology departments offering RC (2.7%) performed more than 50 RCs per year, which increased to 17 of 360 (4.7%) in 2019. In 2019, 107 departments (29%) performed 25–49 RCs and 236 (66%) departments performed < 25 RCs. Distribution of RC patients and number of hospitals performing RC in Germany stratified for annual hospital caseload categories is presented in Fig. 3 (online supplement). The share of patients treated in high-volume hospitals increased from 8.8% to 15.9% from 2006 to 2019 (*p* < 0.001). Figure 4 (online supplement) gives an overview of hospital caseload distribution in Germany in 2006 and 2019, respectively. Table 2 (online supplement) lists the 30 German hospitals performing the highest number of RCs in 2006 and 2019.

## Discussion

This population-based study examined the treatment trends in patients diagnosed with MIBC in Germany between 2006 and 2019. We were able to show, that three out of four patients in Germany diagnosed with bladder cancer stage ≥ T2 receive RC as their primary treatment approach. In Germany, the share of patients receiving no surgical treatment remained low in the present study. In 2006, 8 of 299 urology departments (2.7%) performed more than 50 RCs per year which increased to 17 of 360 (4.7%) in 2019. In 2019, 107 departments (29%) performed 25–49 RCs and 236 (66%) departments performed < 25 RCs.

### Radical cystectomy and adherence to guidelines

Current guidelines in the USA as well as in Europe clearly recommend a radical surgical approach for patients diagnosed with MIBC [3, 4]. Previous studies from the USA investigating the implementation of RC in clinical practice had described low adherence to evidence-based guideline recommendations [12]. Williams et al. described 2016 poor RC results with a share of only 18.9% receiving RC for MIBC [9]. However, the SEER data seem to massively overestimate the share of patients receiving no treatment. Because of this obvious shortcoming we had to waive another planned comparison study of Germany and the USA [13]. For a different aspect, our working group recently compared the German hospital billing database and the Nationwide Inpatient Sample from the USA. We were able to show an increase in the annual numbers of RC treatments in Germany while an annual number of cases in the USA remained stable [14]. In total, the number of RCs in Germany increased by 31% from the years 2006 to 2014 and older patients were the main drivers of growing RC numbers in Germany.

### Surgical approach

RC is an invasive procedure associated with the highest morbidity and mortality in the field of urologic surgery [15, 16]. Different surgical approaches are nowadays available. In Germany classical open RC is the most commonly used approach. Our results showed that 84% of RC in 2019 were performed as open surgery. In the USA, robotic-assisted RC (RARC) has recently gained popularity for patients with MIBC [17]. In 2014, RC was performed robotic-assisted in 20% of cases [18]. Recent studies showed an advantage for RARC in regard to decreased postoperative infections, blood loss as well as the length of hospital stay [17]. Mastroianni et al. showed a significant difference in perioperative transfusion rates (22% vs 41%) in a recent randomized-controlled prospective trial comparing early outcomes of robotic versus open radical cystectomy [19]. In our analysis, only 7% of all RC cases in Germany in 2019 were performed robotic-assisted. Reasons for these low numbers might be the lack of additional reimbursement for the use of a surgical robot due to the principle of cost containment in German healthcare policy [14]. Robotic approach was currently reported as a key driver for the implementation of partial nephrectomy in the USA for the treatment of renal cancer [13]. Thus, RARC might have the potential to increase the share of RC and to support the centralization of surgical care in the USA for the treatment of MIBC.

### Caseload and centralization

In our cohort, around 5% of urological departments in Germany performed more than 50 RCs per year in 2019. Numerous studies have reported on the correlation of caseload volume and perioperative mortality [18, 20]. We showed in the present study, that the share of patients treated in high-volume hospitals increased from 8.8 to 15.9% from 2006 to 2019 (*p* < 0.001). However, 66% of patients who underwent RC in 2019 were treated in a urological department with low volume (< 25 RCs per year). Therefore, there is still further potential for centralization of RC for treatment of MIBC in Germany. Our working group showed a trend of centralization for RC in the United States between 2006 and 2014 [18]. Furthermore, Tuderti et al., recently investigated the impact of a surgeon’s experience on peri-operative and functional outcomes, concluding that patients treated at the beginning of the learning curve showed worse perioperative and functional results. Once the procedure was standardized, complications rates, hospital stay, and day-time continence recovery experienced a significant improvement [21].

### Chemotherapy

Chemotherapy offers an established alternative treatment option for patients with MIBC, which are not qualifying for RC [20]. However, a significant survival advantage is generally only possible in a combination with RC or RT [22, 23].

Our data showed that chemotherapy as a primary single treatment has no important role in the treatment of patients with MIBC. Chemotherapy in a neoadjuvant setting improves survival outcomes for MIBC patients and is recommended by current guidelines [8, 24]. However, due to various reasons such as treatment-related toxicity or treatment at “lower volume” hospitals, there is still an underuse of neoadjuvant chemotherapy [8, 24]. Since recurrence rates after RC remain high between 30 to 45%, multimodality treatment which consists of a combination of surgery with chemotherapy and RT is routinely applied offering more favorable clinical outcomes [22, 25, 26]. We showed in this study, that the combination of RC and chemotherapy is significantly more used in younger patients. Combination of RC and chemotherapy increased from 17% in 2006 to 21% in 2017 for patients younger than 60 years while it only slightly increased from 2% in 2006 to 4% in 2017 for patients older than 80 years.

### Multimodality treatment

Multimodality treatment was stable over the investigated time at around 3% in our analysis. However, the epidemiological cancer registries in Germany are very limited in recording sequential treatments. Therefore, this number is very likely underestimated. A comparative analysis using data from the National Cancer Data Base of the USA with 8379 patients treated between 2004 and 2013 (6606 underwent RC, 1773 underwent primary radiochemotherapy) showed a 5-year overall survival rate of 38% after RC and 30% after primary radiochemotherapy [27].

In the present study, roughly one out of 10 patients received a combination therapy of surgery with either neoadjuvant or adjuvant chemotherapy. In general, in younger patients the share of receiving a combination of RC and chemotherapy was notably higher.

### Radiation therapy

RT is considered as a further option in the treatment of muscle-invasive bladder cancer and is especially used in less fit patients [28]. RT as a single treatment modality is associated with a high number of cases with local recurrence or inadequate first response [29]. Therefore, multimodality treatment with a combination of RT and chemotherapy as well as RC is nowadays commonly recommended [26]. James et al. showed in a prospective randomized trial that simultaneous radiochemotherapy is superior to radiation alone in the treatment of bladder cancer [28].

In Germany, around 1% of patients older than 80 years with MIBC received only RT. In the other age groups, RT as a single treatment approach was not routinely used. Combination of RT with surgery was found in around 4% of patients treated for MIBC while the combination of RT with chemotherapy was neglectable.

### Limitations

While we provide a large retrospective analysis of current treatment trends for MIBC in Germany using the ZfKD data, billing data as well as German hospitals’ quality reports we acknowledge that there are several limitations to this study. A general limitation results from the inferior quality of epidemiological data compared to case records or study files [30]. First, the ZfKD and Destatis databases lack important clinical information on patient characteristics. Moreover, the German ZfKD does not provide details on the specific chemotherapeutic regime used for treatment or if it is applied in an adjuvant or neoadjuvant setting or with palliative intention. However, chemotherapy within 6 months after surgery should be documented according to the rules of the registers. Second, for patients with no active treatment it was not possible to differentiate between missing treatment data or actually no active treatment. Unfortunately, the database does not include separate data on the utilization of newer immunotherapeutic agents for muscle-invasive bladder cancer. Further, there is no information about tumor characteristics. However, since these are the only epidemiological data for Germany, we had to accept these limitations y[11].

Third, the German hospitals’ quality reports database does not allow a combination of ICD and OPS codes. Therefore, a small proportion of RC cases in the institution-related data are not associated with MIBC. This share was stable around 8–10% from 2006 to 2019. Further, there is a shift towards “early cystectomy” for high-risk non-muscle-invasive bladder cancer, which we did not include in the present study. Therefore by including only MIBC the utilization of RC is underestimated.

## Conclusion

This population-based study demonstrates treatment trends for MIBC in Germany over a long study period of 14 years. Most patients undergo RC. Further, multimodality treatment is slightly increasing. The combination of RC with chemotherapy is the most popular multimodality approach. However, neoadjuvant chemotherapy is still underutilized. With overall increasing case numbers of RC, there is a slight tendency toward centralization with 3% of urology departments performing more than 50 RCs per year in 2006 and 5% in 2019, respectively.

## Supplementary Information

Below is the link to the electronic supplementary material.

Supplementary file1 (DOCX 397 kb)

## Data Availability

Luka Flegar had full access to all the data in the study and takes responsibility for the integrity of the data and the accuracy of the data analysis.

## Abbreviations

DRG: Diagnose-related groups
MIBC: Muscle-invasive bladder cancer
RC: Radical cystectomy
RT: Radiation therapy
SEER: Surveillance, Epidemiology, and End Results
US: United States of America
ZfKD: German National Center for Cancer Registry Data
