# Supplementary material for: Treatment trends for muscle-invasive bladder cancer in Germany from 2006 to 2019
Source: World J Urol. 2022 Apr 29;40(7):1715–21. doi: 10.1007/s00345-022-04017-z (PMC9237006; doi:10.1007/s00345-022-04017-z)
Supplement: Supplementary file 1 — Supplementary file1 (DOCX 397 kb) [file 345_2022_4017_MOESM1_ESM.docx]

**Online supplement**

**Table 2.** Overview of performed number (n) of RCs in 2006 and 2019 in Germany (Source: German hospitals’ quality reports).

|  | **2006** | | **2019** | |
| --- | --- | --- | --- | --- |
| **Rank** | **n** | **Place** | **n** | **Place** |
| 1 | 183 | Helios Hospital Salzgitter | 97 | University Hospital TU München |
| 2 | 83 | University Hospital LMU München | 95 | University Hospital TU Dresden |
| 3 | 72 | University Hospital TU Dresden | 95 | University Hospital Mannheim |
| 4 | 72 | University Hospital Ulm | 90 | University Hospital LMU München |
| 5 | 69 | Marien Hospital Herne | 87 | Marien Hospital Herne |
| 6 | 58 | University Hospital Hamburg | 78 | Barmherzige Brüder Hos. München |
| 7 | 57 | Evangelisches Hospital Duisburg | 72 | University Hospital Hamburg |
| 8 | 50 | University Hospital Mainz | 64 | Hospital Barmbek Hamburg |
| 9 | 47 | Hospital Karlsruhe | 63 | Vivantes Auguste-Viktoria Berlin |
| 10 | 46 | University Hospital Berlin | 62 | University Hospital Münster |
| 11 | 46 | Hospital St.Georg Leipzig | 61 | Hospital Fulda |
| 12 | 46 | Hospital Barmbek Hamburg | 60 | Caritas-Hospital Regensburg |
| 13 | 44 | Barmherzige Brüder Hos. Trier | 59 | Vivantes Humboldt Berlin |
| 14 | 44 | Hospital Oldenburg | 57 | University Hospital Köln |
| 15 | 42 | Hospital Kassel | 52 | Hospital Lippe Detmold |
| 16 | 42 | Asklepios Hospital Harburg | 50 | Diakonie-Hospital Stuttgart |
| 17 | 42 | Hospital Essen | 50 | Hospital Nord Dortmund |
| 18 | 41 | Malteser Hospital Bonn | 49 | Hospital Siloah Hannover |
| 19 | 41 | Caritas-Hospital Regensburg | 49 | Vivantes Am Urban Berlin |
| 20 | 40 | University Hospital Essen | 48 | University Hospital Freiburg |
| 21 | 40 | University Hospital Kiel | 46 | University Hospital Rostock |
| 22 | 40 | Hospital Nord Dortmund | 46 | Hospital München - Planegg |
| 23 | 39 | Vivantes Auguste-Viktoria Berlin | 45 | University Hospital Ulm |
| 24 | 39 | Hospital Augsburg | 45 | University Hospital Augsburg |
| 25 | 39 | Hospital München - Planegg | 45 | University Hospital Leipzig |
| 26 | 39 | Franziskus Hospital Berlin | 45 | University Hospital Tübingen |
| 27 | 38 | University Hospital Münster | 45 | Loretto-Hospital Freiburg |
| 28 | 37 | Hospital München-Bogenhausen | 45 | Hospital Bremen-Mitte |
| 29 | 37 | Königin-Elisabeth-Hospital Berlin | 45 | Hospital Essen-Hiltrup |
| 30 | 36 | Paracelsus-Hospital Düsseldorf | 44 | University Hospital Mainz |

**Fig. 2.** Share of treatment trends for muscle-invasive bladder cancer in Germany from 2006 to 2017 in percent for different age groups (<60y; 60-79y; >80y) (0% from 2006-2017: Chemotherapy, RT and Chemo) (Source: German National Center for Cancer Registry Data).

**Fig. 3.** Distribution of RC patients (a) and number of hospitals performing RC (b) in Germany stratified for annual hospital caseload categories from 2006 to 2019 (Source: Nationwide hospital billing database of the German Federal Statistical Office (Destatis database)).

**~~
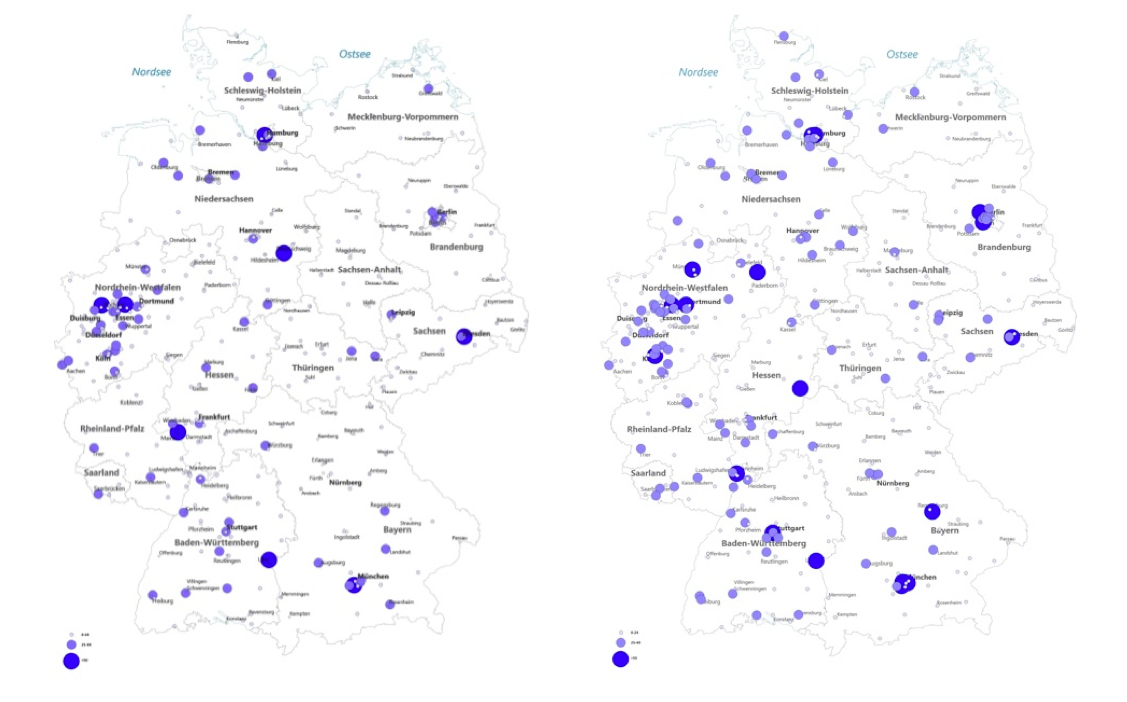
~~**

**Fig. 4.** RC in Germany in 2006 (left) and 2019 (right) (0-24, 25-49, >50 performed RC) (Source: German hospitals’ quality reports).
